# Plant–Insect Interactions in Biological Invasions: From Classical Hypotheses to Multitrophic Networks and Biological Control

**DOI:** 10.3390/insects17100989

**Published:** 2026-09-25

**Authors:** Qinchun Li, Fanping Xu, Wenzhi Cheng, Chengmei Qi, Xiaowen Liu

**Affiliations:** 1 College of Civil and Environmental Engineering, Hunan Institute of Science and Engineering, Yongzhou 425199, China; lqc0657@126.com; 2 College of Chemistry and Bioengineering, Hunan Institute of Science and Engineering, Yongzhou 425199, China; xufanping2024@163.com; 3 School of Information Engineering, Hunan Institute of Science and Engineering, Yongzhou 425199, China; chengwenzhi@huse.edu.cn

**Keywords:** enemy release hypothesis, novel weapons hypothesis, evolution of increased competitive ability, food web, trophic cascade, pest management

## Abstract

Biological invasions are a critical driver of global environmental change, with plant–insect interactions central to invasion success and biological control. This review systematically examines three classical hypotheses—the Enemy Release Hypothesis (ERH), the Novel Weapons Hypothesis (NWH), and the Evolution of Increased Competitive Ability (EICA) hypothesis, within a plant–insect context. Our synthesis shows that ERH support is widespread but highly contingent on environmental context; NWH highlights the negative effects of invasive allelochemicals on native insects and soil biota; and the EICA hypothesis provides an evolutionary framework for understanding shifts in defence–growth resource allocation in invasive plants. We advocate for a multitrophic network perspective that captures ecosystem complexity and elucidates synergistic interactions among hypotheses across space and time. We identify inherent limitations in single-hypothesis biocontrol approaches and propose an integrative management framework grounded in network theory. Emerging tools—including molecular ecology and network modelling—are emphasised for resolving cryptic interactions and forecasting control outcomes. Future priorities include interdisciplinary integration, long-term monitoring, global comparisons, and addressing climate-driven uncertainties. This review aims to advance both theoretical invasion ecology and the development of sustainable, evidence-based biological control strategies.

## Simple Summary

Biological invasions drive biodiversity loss, ecosystem degradation, and substantial economic costs globally. A key question is why a few introduced plants become invasive while others fail. This review examines three main explanations centred on plant–insect interactions: escape from natural enemies, production of novel suppressive chemicals, and evolutionary shifts from defence to growth. Our synthesis shows these mechanisms often interact synergistically, with their importance varying across species, communities, and environments. We argue that understanding invasions requires moving beyond pairwise interactions to embrace entire food webs, including soil biota, herbivores, and their natural enemies, as invasions trigger cascading effects across trophic levels. From this integrated perspective, we propose a biological control framework that prioritises local food-web integrity, complementary natural enemy deployment, and the use of molecular tools and predictive models for improved risk assessment. This review provides researchers and land managers with a practical, evidence-based framework for developing sustainable invasive species management strategies under current and future environmental change.

## 1. Introduction

Globalization-driven human-mediated species exchanges have intensified biological invasions into a critical environmental issue under global change [1,2]. A comprehensive database of 45,813 first records across 16,926 established alien species reveals that the global annual introduction rate has risen steadily over the past two centuries, with 37% of all records concentrated in 1970–2014 and no saturation evident for most taxa, underscoring that past mitigation measures have lagged far behind the pace of globalization [1]. Invasive plants, through competition, allelopathy, and habitat modification, profoundly affect native plant communities and their dependent biota [3], while also altering nutrient cycling, hydrology, and disturbance regimes, thereby impairing ecosystem services; these effects are further amplified by synergies with other global change drivers [2]. Nevertheless, the mechanistic basis of successful plant invasion, particularly the multifaceted roles of secondary metabolites in allelopathic interactions, defence responses, soil microbial feedbacks, and resource competition remains inadequately understood [3]. Elucidating these metabolite-mediated invasion strategies therefore constitutes a central research priority in invasion ecology and provides a critical scientific foundation for integrated weed management.

Plant–insect interactions constitute a foundational interspecific relationship in terrestrial ecosystems, underpinning food-web architecture, energy flux, nutrient cycling, and coevolutionary dynamics. In the context of biological invasions, insect herbivores and their natural enemies are pivotal biotic determinants of the colonization, establishment, and spread of invasive plants [4]. The interplay between invasive plants and native herbivores, and its cascading effects on higher trophic levels (e.g., parasitoids), represents a core mechanism shaping invasion dynamics and their ecological consequences [5]. Furthermore, the spread of invasive plants is tightly coupled with insect invasions: non-native plants can create novel niches for obligate phytophagous insects, thereby facilitating secondary insect invasions, a pattern supported by macroecological evidence showing that plant proliferations are major drivers of insect invasions [6]. The Enemy Release Hypothesis (ERH) posits that invasive plants succeed largely because they escape co-evolved specialized natural enemies in their native ranges [7]; however, recent work also demonstrates that release from generalist herbivores, such as granivorous rodents, can substantially enhance invasion success, challenging the traditional focus on specialists alone [7]. Yet ERH typically emphasises direct plant–enemy interactions, often overlooking broader community contexts. In practice, invasive plants may compete with natives for pollinators, alter herbivore feeding preferences, and provide novel habitats and resources for both herbivores and their natural enemies, thereby rewiring entire food webs and parasitoid–host association networks [5,8]. Insects thus assume multiple, sometimes contradictory, roles in the ecological and evolutionary processes underpinning plant invasion success: as herbivores, they impose selective pressures driving defensive trait evolution; as pollinators, they influence sexual reproduction and gene flow; and as natural enemies, they may suppress invasions or, paradoxically, promote them by disrupting native enemy networks [6]. Long-term experiments further reveal that the facilitative effects of above- and belowground insect herbivores on invasive plants can intensify over years, underscoring the need for sustained observations to capture invasion trajectories [4]. Consequently, elucidating the multifaceted interactions between invasive plants and insects is not only central to understanding invasion mechanisms, but also critical for informing effective biological control strategies.

Biological invasions have become a primary driver of global biodiversity loss and ecosystem functional degradation, incurring substantial economic and ecological costs annually [9]. To elucidate the mechanisms underlying invasion success, ecologists have proposed a range of hypotheses over the decades, among which the ERH [10], the novel weapons hypothesis [11], and the Evolution of Increased Competitive Ability (EICA) hypothesis [12] are particularly influential. Although these hypotheses all involve, to varying degrees, plant–insect interactions, traditional research has largely focused on testing a single hypothesis or examining pairwise species interactions (e.g., invasive plant–specialist herbivore, parasitoid–invasive insect), often lacking a systematic framework that integrates horizontal comparisons and vertical syntheses across hypotheses [13]. Moreover, linking population and community ecology with individual-level variation in insect physiology and behaviour across trophic levels remains a critical but underexplored avenue [14].

Accumulating empirical evidence has revealed the limitations and ongoing controversies surrounding each of these hypotheses [15], and the mechanisms they describe are not mutually exclusive; they may operate simultaneously, interact, and even amplify one another during actual invasion processes. Furthermore, the impacts of invasions are not transmitted linearly along food chains but instead propagate across multiple trophic levels through complex indirect effects and feedback loops [16]. Invasive plants not only interact directly with insect herbivores but also indirectly affect belowground insect populations via interactions with soil biota, such as mycorrhizal fungi and pathogens [17,18]. Similarly, parasitoids (key agents of biological control) are strongly influenced by host plants, herbivores, and pathogenic microorganisms (including parasitoid-associated viruses) in their interactions with hosts [5,19]. This intricate network comprising plants, herbivores, natural enemies, and microbes necessitates a multi-trophic network perspective on invasion processes. Consequently, re-evaluating classical hypotheses and promoting their integration with multi-trophic network theory is not only a theoretical imperative for invasion ecology but also offers practical guidance for designing effective biological control strategies.

## 2. Retrospection, Verification and Contemporary Revaluation of the Classical Hypothesis

### 2.1. Enemy Release Hypothesis

#### 2.1.1. Theoretical Framework and Mechanism of Action

The Enemy Release Hypothesis (ERH) remains one of the most influential paradigms in invasion ecology [10]. It posits that upon introduction to a region outside their native range, plants escape the regulatory effects of co-evolved, host-specific natural enemies, primarily phytophagous insects and pathogenic microorganisms, that exert strong top-down control in their original habitats. Liberated from these constraints, invasive plants are able to reallocate resources previously invested in defence towards growth and reproduction, thereby acquiring a competitive advantage over native species and ultimately driving population outbreaks [20]. This resource-allocation trade-off, often manifesting as reduced herbivore damage and altered structural defences in invaders [20], underpins the ERH’s mechanistic core. The hypothesis also provides the theoretical foundation for classical biological control, which aims to re-establish suppression of invasive plants by introducing their co-evolved natural enemies from the native range [13]. Importantly, the ERH logic extends beyond plant–herbivore interactions to animal invasions: the absence of parasites, pathogens, or other natural enemies is similarly considered to facilitate the establishment and spread of invasive insect species, as documented in recent analyses of invasive *Vespa velutina* (Lepeletier, 1836) (Hymenoptera: Vespidae) and its parasite prevalence relative to native Hymenoptera [21].

#### 2.1.2. Empirical Support and Empirical Controversy in Plant–Insect Interactions

Since the proposition of the Enemy Release Hypothesis (ERH), a substantial body of empirical work has accumulated, providing considerable support across diverse taxa and ecosystems. In a systematic comparative study involving 47 pairs of invasive and native plants in China, Huang et al. [20] found that invasive plants suffered significantly lower leaf herbivore damage and exhibited weaker structural defences compared with co-occurring native or non-invasive alien plants, consistent with the predicted resource-allocation shift. In plant–soil systems, Guerrero et al. [18] reported that the abundance of phytophagous nematodes in the rhizosphere of the invasive *Pinus elliottii* Engelm. (Pinaceae) was markedly lower than that associated with native tree species, indicating belowground enemy release. Field experiments have provided direct mechanistic evidence: Lucero et al. [7] demonstrated that granivorous rodents reduced seedling establishment of *Bromus tectorum* L. (Poales: Poaceae) by nearly 60% in its native range (western Asia), whereas no such effect occurred in the invaded Great Basin Desert, confirming that escape from generalist seed predators can facilitate invasion. In the context of insect invasions, Walsh-Antzak and Erickson [22] found that larvae of the invasive African fig fly *Zaprionus indianus* (Gupta, 1970) (Diptera: Drosophilidae) gained a competitive advantage in the presence of parasitoids, but only under high-density conditions, suggesting that enemy release can be context-dependent. Similarly, Hartshorn et al. [23] showed that the invasive *Pyrus calleryana* Decne (Rosales: Rosaceae) experienced substantially less damage from specialist herbivores than did native tree species, further corroborating the ERH. The ERH logic has also been extended to animal invasions. Bartolomé et al. [21] documented significantly lower prevalence of parasites and pathogens in the invasive hornet *V. velutina* compared with native Hymenoptera, aligning with predictions. In freshwater turtles, Rocha et al. [24] observed that the detection rate of parasitic leeches on invasive *Trachemys* turtles was only 1/18 of that on native turtles over a 10-year co-occurrence period, with some invasive individuals entirely free of infestation.

Despite these supportive lines of evidence, the ERH has encountered substantial challenges and qualifications. Several studies have indicated that invasive plants do not necessarily completely escape their natural enemies; instead, they may accumulate generalist or omnivorous natural enemies in their introduced ranges. Dickie et al. [25] proposed that even when invaders suffer substantial herbivory, they can offset such losses through rapid growth rates, and the resulting “enemy spillover” may indirectly harm native plants, thereby reinforcing invader dominance, a nuanced counterpoint to the classic ERH. Moreover, the strength of enemy release is strongly contingent on environmental context. For example, Walsh-Antzak and Erickson [22] observed that the benefit of release from parasitoids for *Z. indianus* was density-dependent, with no detectable effect at low densities but a significant advantage at high densities. An eight-year field experiment by Korell et al. [4] on the invasive plant *Solidago canadensis* L. (Asteraceae) revealed that above- and belowground insect herbivores did not suppress, but rather promoted, the invader’s establishment and growth by preferentially feeding on native competitors, highlighting the importance of indirect community-level effects that the ERH often overlooks. In vertebrates, Roznik et al. [26] conducted a field removal experiment on parasites of the invasive Cuban tree frog *Osteopilus septentrionalis* (Duméril & Bibron, 1841) (Anura: Hylidae) and found no significant effect on growth or survival, suggesting that enemy release was not the primary driver of its invasion success. For insect parasitoids, Ricupero et al. [27] conducted a global survey of the ladybeetle parasitoid *Dinocampus coccinellae* (Schrank, 1802) (Hymenoptera: Braconidae) and found strikingly similar parasitism rates (approximately 4–7%) between the invasive *Harmonia axyridis* (Pallas, 1773) (Coleoptera: Coccinellidae) and native ladybirds, contradicting the ERH prediction. Collectively, these contradictions underscore the necessity of re-evaluating the ERH within a broader multitrophic and context-dependent framework, and of integrating its predictions with alternative hypotheses that account for indirect interactions, trait-mediated effects, and environmental heterogeneity.

#### 2.1.3. Challenges and Reinterpretations of Modern Research

Recent empirical and theoretical advances have challenged the classical ERH framework along three interconnected axes: the overlooked role of omnivorous natural enemies, the neglect of indirect community-level interactions, and the strong context dependence of enemy release effects.

Firstly, the assumption that invasive plants escape natural enemies entirely has been complicated by the rapid exploitation of invaders by generalist or omnivorous consumers in the introduced range. Dickie et al. [25] emphasised that while invasive plants may lose their co-evolved specialists, they are often readily consumed by resident generalist herbivores, which may preferentially feed on invaders due to their favourable traits, such as higher nutritional quality and lower structural defences. Paradoxically, this consumption can generate “enemy spillover” onto native plants, indirectly reinforcing invader dominance rather than suppressing it.

Secondly, the ERH has traditionally focused on pairwise interactions, largely overlooking the indirect effects that natural enemies exert through community-level trophic networks. Korell et al. [4] demonstrated that above- and belowground insect herbivores did not suppress the invasive *S. canadensis* L., but instead facilitated its establishment by preferentially feeding on native competitors, thereby altering competitive hierarchies in ways not predicted by a simple enemy-release framework. Similarly, cascading effects mediated by plant pathogens can decouple trophic transmission. Albittar et al. [19] found that Beet yellows virus infection altered host plant quality and aphid performance, yet these effects did not propagate to the parasitoid third trophic level, suggesting that the benefits of enemy release may be attenuated or blocked across trophic linkages.

Thirdly, the magnitude and even the direction of enemy release are strongly contingent on environmental and biotic context. Temperature, humidity, host density, and the composition of competitor communities can all modulate the strength of natural enemy effects. The invasive ants, *Linepithema humile* (Mayr, 1868) (Hymenoptera: Formicidae) provide a compelling illustration: during invasion, the diversity of associated microbial communities collapsed dramatically (from 120 to 57 genera) due to serial population bottlenecks, representing a form of microbial enemy release that contributes to invasion success. However, in different introduced regions (e.g., the United States vs. New Zealand), these ants subsequently acquired novel symbionts, revealing that enemy release and pathogen accumulation are not mutually exclusive but occur as dynamic, stage-specific processes [28]. Likewise, Withers et al. [29] investigated the microbial pathogens of the invasive fall armyworm *Spodoptera frugiperda* (J.E. Smith, 1797) (Lepidoptera: Noctuidae) in Africa and found that within just three years of invasion, the pest had acquired both co-introduced pathogens from its native range and novel pathogens that “spilled over” from local insect reservoirs (e.g., African noctuid moths). This coexistence of enemy release and enemy spillover underscores that these processes operate simultaneously, rather than as alternative endpoints.

In sum, the ERH remains a valuable conceptual cornerstone for understanding invasion success, but it must be reframed within a broader, dynamic ecological and evolutionary context. Rather than equating enemy release with the simple absence of local natural enemies, it should be viewed as a conditional and transient process whose net outcome depends on the interplay between the loss of specialist enemies, the accumulation of generalist consumers, indirect community interactions, and environmental heterogeneity [30]. Such an integrative perspective not only refines the hypothesis but also enhances its predictive power for invasive species management and biological control strategies.

### 2.2. Novel Weapons Hypothesis

#### 2.2.1. Theoretical Basis of Allelopathy and Chemical Defence

The Novel Weapons Hypothesis (NWH) proposes that certain invasive plants gain a competitive advantage in recipient communities by producing allelochemicals to which native species have not been evolutionarily exposed. Lacking co-evolved tolerance or detoxification mechanisms, natives are disproportionately suppressed, whereas the invaders themselves exhibit constitutive resistance to their own compounds. At its core, the NWH thus relies on biochemical novelty and the resultant non-coevolutionary interactions [31]. Although originally conceived to explain plant–plant allelopathy, the hypothesis has been extended to encompass effects on insect herbivores, soil fauna, and microbial communities [32,33]. For instance, allelopathic root exudates can directly inhibit germination and seedling growth of natives, while also impairing the physiology of non-target soil organisms such as earthworms and reducing overall soil microbial activity, thereby amplifying invasion impacts through multiple belowground pathways [32,33].

The invasive *Tithonia diversifolia* (Hemsl.) A. Gray (Asteraceae) which forms dense monodominant stands in both its native Central American range and invaded regions of Southeast Asia, Africa, and southern China, exemplifies the dual functionality of novel chemical weapons. Kato-Noguchi and Kato [34] showed that its secondary metabolites serve concurrently as anti-herbivore defences and as allelopathic agents against neighbouring competitors. Likewise, the chemical arsenal of *Lantana camara* L. (Verbenaceae) contributes to invasion success via combined roles in defence, allelopathy, and insect resistance [35]. Collectively, these multi-functional chemical systems represent a key adaptive trait underpinning the establishment and spread of invasive plants in novel environments.

#### 2.2.2. Effects on Insect Herbivores

Allelochemicals produced by invasive plants influence multiple dimensions of insect herbivore performance, including feeding preference, growth and development, survival, and population dynamics. These effects may be direct (for instance, through toxicity or antinutritive properties) or indirect, via alterations in host plant nutritional quality or the induction of defensive responses. Liu et al. [32] demonstrated that root and leaf extracts from three widespread invasive Asteraceae species, *Ageratina adenophora* (Spreng.) R.M.King & H.Rob. (Asteraceae), *Bidens bipinnata* L. (Asteraceae), *Erigeron annuus* (L.) Pers. (Asteraceae), exerted pronounced toxic effects on the model soil organism *Eisenia fetida* (Savigny, 1826) (Haplotaxida: Lumbricidae), inducing weight loss, respiratory depression, and oxidative damage. The toxic effects of these invasive plant extracts were significantly stronger than those of native plant extracts. This finding indicated that allelochemicals can transcend trophic boundaries and directly affect soil decomposers, thereby influencing broader ecosystem processes. Similarly, Qu et al. [33] reported that root extracts of *Rhus typhina* L. (Anacardiaceae) not only suppressed the growth of the native plant *Tagetes erecta* L. (Asteraceae), reducing plant height, root length, and biomass, but also inhibited soil microbial activity and decreased the utilisation efficiency of carbon sources such as carbohydrates and carboxylic acids. Such suppression of soil microbial communities may indirectly compromise herbivore fitness by limiting access to beneficial symbionts, including nitrogen-fixing bacteria.

Beyond allelopathic effects on plants and soil biota, several invasive species produce volatile or exudate compounds with direct insecticidal or nematicidal activity. For instance, *T. diversifolia* releases α-pinene and the nematicidal sesquiterpene tirotundin [34], while *L. camara*, a globally recognised invasive species, produces an array of volatile organic compounds with documented insecticidal properties [35]. These multi-functional chemical defences collectively reinforce the competitive dominance of invaders and extend the explanatory scope of the NWH beyond plant–plant interactions to encompass insect herbivores and their natural enemies [31].

However, the effects of invasive plant chemicals on insects are not uniformly negative. Some herbivores have evolved the capacity to exploit these compounds for their own defence. Villanueva-Hernández and Núñez-Farfán [36] observed that larvae of the specialist beetle *Lema daturaphila* (Kogan & Goeden, 1970) (Coleoptera: Chrysomelidae), which feeds on *Datura stramonium* L. (Solanaceae), secrete oral secretions containing host-plant alkaloids when attacked by parasitoid flies, effectively co-opting the plant’s chemical defences against their own natural enemies. This adaptive strategy illustrates a novel facet of plant–insect interactions in invaded systems: rather than merely repelling herbivores, invader allelochemicals may inadvertently provide protection to specialised consumers, thereby creating complex, indirect effects that cascade through trophic networks. Such counter-intuitive outcomes underscore the need for a multi-trophic perspective when evaluating the ecological consequences of novel chemical weapons in biological invasions.

#### 2.2.3. Key Empirical Tests and Applicability Limitations

Despite considerable experimental support, the NWH faces several critical limitations that warrant careful scrutiny. A primary concern pertains to the concentration-dependency of allelopathic effects, which raises questions about the ecological realism of laboratory assays that often employ artificially high extract concentrations [31]. Effah and Clavijo McCormick [31] illustrated this issue by testing aqueous root extracts of the invasive *Cytisus scoparius* (L.) Link (Fabaceae) (Scotch broom) and *Calluna vulgaris* (L.) Hull (Ericaceae) (heather) on the germination and seedling growth of the New Zealand native *Leptospermum scoparium* J.R.Forst. & G.Forst. (Myrtaceae) (mānuka). While invasive plant extracts significantly inhibited mānuka germination across all tested concentrations (0.1–100%), adverse effects on seedling growth and biomass were observed only at higher concentrations (≥5%). Moreover, broom extracts exerted stronger allelopathic effects than heather extracts at the highest concentration (100%), whereas extracts from conspecifics or other native species (e.g., *Chionochloa rubra* Zotov (Poales: Poaceae)) produced only weak inhibition at very high concentrations (50–100%). Importantly, allelopathic inhibitory effects are highly context-dependent, meaning that conclusions drawn from a given region or site cannot be universally generalized to other environments. Realized allelopathic impacts are shaped by soil properties, local microbial communities, and interspecific variation in plant tolerance, rather than constituting a fixed, intrinsic trait of the allelochemical producing donor plant. We accordingly advocate for more rigorous field experiments that manipulate allelochemical concentrations under natural conditions and incorporate biotic and abiotic moderators, rather than relying exclusively on laboratory-based assays.

Second, the NWH predominantly focuses on direct plant–plant interference, leaving multi-trophic and indirect effects largely underexplored. Allelochemicals may inadvertently affect non-target organisms, including pollinators, soil decomposers, and root-associated microbiomes, thereby generating cascading ecological consequences that extend well beyond pairwise competitive interactions. Furthermore, the hypothesis does not operate in isolation; it frequently intersects with other invasion mechanisms, most notably the ERH. Nakamura et al. [37] demonstrated that leaf pathogenic fungi were more abundant in the native Japanese range of *Ardisia crenata* Sims (Primulaceae) than in its exotic Florida range (a pattern consistent with ERH) while also revealing that allelochemicals can reshape leaf and root microbial community composition, potentially modulating pathogen pressures in ways that either reinforce or counteract NWH predictions. This interplay highlights the necessity of examining multiple hypotheses within a unified ecological framework rather than treating them as mutually exclusive alternatives.

Third, a recent systematic reassessment has raised more fundamental concerns about the NWH, including definitional ambiguities and insufficient empirical evidence to support its core predictions [15]. Caution is warranted when interpreting laboratory-based assays of enemy release; field validation across different regions and plant organs is essential before drawing generalis conclusion. Many published studies fail to establish causal relationships between allelochemical synthesis and plant-fitness metrics or population growth under natural conditions. Therefore, there is an urgent need for rigorous field experiments that integrate competitive results, resource availability, and adaptive evolution. From this critical point of view, NWH is still instructive and useful, but should not be invoked in isolation. Its predictive power and explanatory scope can only be achieved by embedding it into a broader multi-hypothesis framework, taking into account direct and indirect biological interactions, environmental heterogeneity, and evolutionary dynamics across trophic levels.

### 2.3. Evolution of Increased Competitive Ability Hypothesis

#### 2.3.1. Trade-Off Hypothesis and Defence–Growth Differentiation Mechanism

The Evolution of Increased Competitive Ability (EICA) hypothesis extends the ERH by incorporating an evolutionary dimension. It posits that upon introduction to a novel range, invasive plants escape the selective pressure exerted by co-evolved specialist natural enemies, particularly insect herbivores, thereby rendering the high-cost defensive traits previously maintained in the native range superfluous. Consequently, natural selection favours genotypes that reallocate resources from defence to growth and reproduction, leading to invasive populations that exhibit enhanced competitive ability in resource acquisition. In essence, the EICA hypothesis predicts an evolutionary shift from a “defence-oriented” to a “competition-oriented” life-history strategy, underpinned by a fundamental trade-off between defence investment and growth [20].

Plant defence against insect herbivory encompasses a diverse array of strategies, broadly categorised into physical barriers, chemical defences, ecological regulation (e.g., indirect defence via volatile emission), and behavioural or induced responses [38]. In invasive plants, the relaxation of pressure from specialist natural enemies is expected to reduce allocation to these defensive traits, liberating resources for increased biomass production, rapid growth, and greater reproductive output, thereby conferring a competitive advantage over native species. However, meta-analytical evidence has provided only partial support for the EICA framework. While invasive populations do exhibit consistently higher productivity than their native conspecifics, the predicted concomitant reduction in defence, and the corresponding trade-off between defence and competitive ability, has not been widely corroborated across taxa [39]. This discrepancy suggests that the defence–growth trade-off may be more context-dependent than originally proposed, potentially modulated by resource availability, enemy pressure in the introduced range, or the evolutionary history of the invader. These unresolved patterns underscore the need for more nuanced, multi-species investigations that integrate physiological, ecological, and evolutionary perspectives to fully evaluate the EICA hypothesis.

#### 2.3.2. Plant Adaptive Evolution Under Insect Selection Pressure

As the primary guild of herbivorous natural enemies, insects exert strong selective pressures that drive adaptive evolution in invasive plants. The EICA hypothesis predicts that relaxation of this pressure in novel ranges should result in reduced allocation to defensive secondary metabolites and increased investment in growth and reproductive traits. Empirical evidence, however, remains equivocal. In a large-scale comparative study, Huang et al. [20] found that invasive plants exhibited lower structural defence investment, including reduced leaf density, cellulose, and lignin content, alongside higher nutrient concentrations (e.g., nitrogen) relative to native species. This “less defence, more growth” phenotypic pattern aligns with EICA predictions. Nevertheless, patterns of chemical defence are more nuanced. Cheng et al. [40] examined pyrrolizidine alkaloid diversity and total concentrations in Senecio vulgaris populations and found only a non-significant tendency towards reduced alkaloid content in invasive populations, providing only partial support for the predicted overall decline in chemical defence. This suggests that some invaders may adopt alternative chemical defence strategies, such as qualitative shifts in toxin composition, rather than simply reducing total investment.

Evidence from animal invasions offers complementary insights. Mühlenhaupt et al. [41] reported that invasive toads on Mauritius and Réunion, where natural enemy diversity is low, exhibited significantly reduced venom-gland size and altered toxin profiles, consistent with both ERH and EICA predictions. In contrast, toads in Cape Town, South Africa, where a diverse enemy community persists showed changes in toxin composition but no reduction in gland size, indicating that the strength of evolutionary responses is modulated by local selection regimes. Similarly, Gastineau et al. [42] detected only low-prevalence infections of the microsporidian genus *Mitosporidium* (Haag et al., 2015) (Rozellomycota) in invasive land flatworms, with markedly lower parasite richness than native-range populations. This pattern supports the idea that parasite release (i.e., enemy release) may allow invaders to reduce immune investment and reallocate energy to other fitness-related traits.

Plant–insect dynamics in invaded ranges can also differ markedly from native ranges. Sakata et al. [43] observed that population densities of the herbivore *Corythucha marmorata* (Uhler, 1878) (Hemiptera: Tingidae) were higher on *Solidago altissima* L. (Asteraceae) in the introduced range than in the native range, and that the presence of this natural enemy actually enhanced the plant’s biotic resistance. This counter-intuitive finding highlights that enemy release does not uniformly reduce herbivore pressure; rather, the direction and magnitude of selection on defensive traits depend on the identity, abundance, and impact of natural enemies encountered in the introduced community. Collectively, these studies underscore that EICA predictions are not universally applicable but are contingent upon the specific ecological and evolutionary context of each invasion event.

#### 2.3.3. Current Research Progress and Future Directions

Contemporary research on the EICA hypothesis has moved beyond the simplistic “input-output” framework, embracing more nuanced and integrative approaches that consider the context-dependent nature of plant defence evolution.

Firstly, plant defence responses are increasingly recognised as heterogeneous rather than uniform. The predicted reduction in defence under EICA may manifest primarily as qualitative defence against specialist natural enemies (e.g., changes in specific toxin profiles), whereas quantitative defences against generalist herbivores (such as leaf toughness or trichome density) may remain unchanged or even increase. Shi et al. [44] demonstrated that the oriental armyworm *Mythimna separata* (Walker, 1865) (Lepidoptera: Noctuidae) selectively oviposits on senescent leaves to avoid triggering plant defence responses, including the jasmonic acid signalling pathway. This behavioural adaptation suggests that insect natural enemies can exert strong selective pressure even in introduced ranges, driving the evolution of sophisticated defence-avoidance strategies in invasive plants. Consequently, rather than a wholesale reduction in defence, plants may enhance specific defensive traits when particular herbivores become dominant threats. This dynamic interplay between enemy escape (as posited by ERH), resource reallocation (as posited by EICA), and novel selective pressures generates complex and often non-additive phenotypic outcomes.

Secondly, future EICA research must incorporate abiotic drivers. Climatic factors, including temperature and CO_2_ concentration, can modulate the induced expression of plant defences and thereby alter herbivore pressure, with direct relevance to the defence–growth trade-off central to EICA. Munawar et al. [45] showed that heat stress altered volatile emissions in potato, reducing attractiveness to pests while increasing attraction to their parasitoids. Such findings indicate that under global climate change, environmental stress may reshape plant–insect chemical communication and thus mediate the defence–growth trade-offs assumed by the EICA hypothesis [46]. Incorporating these abiotic modulators is essential for predicting invasion outcomes under future climatic scenarios.

Thirdly, an important frontier lies in integrating the EICA hypothesis with multitrophic network theory to examine how defence reconfiguration affects entire communities. Shifts in invasive plant defence strategies not only influence herbivore performance but may also propagate through food webs to affect higher trophic levels, such as parasitoids, thereby restructuring community composition and interaction networks. Therefore, future studies should prioritise long-term, multi-factorial experiments that simultaneously evaluate the EICA hypothesis alongside the ERH and NWH, in order to disentangle their relative contributions and synergistic effects on ecosystem functioning [47]. Such an integrative framework will be critical for advancing invasion theory and informing evidence-based management strategies.

### 2.4. Theoretical Relevance and Integrated Analytical Framework of Multiple Hypotheses

The three classical hypotheses outlined above are not mutually exclusive but rather mechanistically interconnected and ecologically nested, jointly shaping the trajectory of biological invasions. In order to facilitate comparison, we summarize the core predictions, empirical support and limitations of the three hypotheses (Table 1).

The ERH can be viewed as a prerequisite for the EICA hypothesis: without release from co-evolved natural enemies, the evolutionary reallocation of resources from defence to growth would not be favoured. The NWH, in turn, provides an additional chemical competitive advantage that operates independently of enemy release, potentially amplifying the benefits of ERH, even when local natural enemies remain present. For instance, an invasive plant may simultaneously escape its native specialists (ERH), evolve enhanced competitive ability through resource reallocation (EICA), and produce novel allelochemicals that suppress both herbivores and neighbouring plants in the introduced range (NWH) [30]. These mechanisms can act synergistically, endowing invasive species with extraordinary competitive superiority that far exceeds what any single hypothesis alone would predict.

A compelling illustration of this mechanistic interplay comes from Shen et al. [48], who investigated secondary invasion dynamics following biological control of the invasive plant *Ambrosia artemisiifolia* L. (Asteraceae). After successful suppression of the primary invader through introduced natural enemies, other invasive plants, including species from the Asteraceae and Poaceae families, rapidly expanded and occupied the vacated niche. The intensity of this secondary invasion was initially governed by phylogenetic relatedness to the target invader: species with close genetic relationships and thus similar defence mechanisms were more likely to succeed, indirectly supporting the kinship-based enemy release hypothesis (i.e., shared natural enemies among closely related species). Subsequently, secondary invasion success became more strongly associated with functional traits (such as plant height and specific leaf area) that are central to resource competition, aligning with predictions of the EICA hypothesis. Moreover, if secondary invaders also possessed allelopathic properties, the NWH further compounded their competitive advantage. This case clearly demonstrates that no single hypothesis can adequately explain complex invasion dynamics; only by integrating multiple hypotheses within a unified theoretical framework can we achieve more accurate predictions and effective management interventions.

Consequently, future research urgently needs to move beyond a “hypothesis-centric” or oppositional mindset towards a “hypothesis-synthesis” approach. This requires designing well-structured experiments that quantify the relative contributions of different mechanisms and their interactions within specific invasion systems. In particular, given the intricate networks comprising plants, herbivores, natural enemies, and soil microorganisms, it is essential to transcend the limitations of single-hypothesis testing and foster genuine dialogue and integration among theoretical frameworks. This integrative imperative is precisely the focus of the following sections. Importantly, all three hypotheses converge on a common axis: they depend critically on plant–insect interactions. Therefore, to understand their synergies and contingencies, these interactions must be embedded within a broader, more structured multitrophic network perspective—an approach that promises to deepen our mechanistic understanding of invasions and to enhance the predictive power of invasion ecology.

In summary, the ERH, NWH, and EICA are not mutually exclusive competing hypotheses but rather generate synergistic amplification effects through three pathways—niche release, chemical suppression, and evolutionary reallocation—that collectively drive invasion success. To visualise the logical relationships and interactive mechanisms among the three hypotheses, we constructed a conceptual diagram of their synergistic framework (Figure 1). As shown in Figure 1, the overlapping areas among the hypotheses represent potential nodes of synergy, while peripheral modulating factors—invasion stage, environmental conditions, species traits, and community context—determine the relative contribution of each hypothesis. This integrative framework provides a theoretical foundation for understanding invasion dynamics from a multitrophic network perspective in the following sections.

## 3. Reconstruction of Invasion Ecology from a Multitrophic Network Perspective

### 3.1. Conceptual Framework and Core Analytical Approaches in Network Ecology

Traditional invasion ecology has largely focused on pairwise interactions between invasive plants and single native species or individual trophic levels (such as plant–herbivore or plant–pollinator relationships) thereby failing to capture the full complexity of invasion impacts on ecosystem structure and function. The multitrophic network perspective offers a paradigm shift by transcending binary interactions and conceptualising all ecological relationships, including feeding, parasitism, mutualism, and competition, across trophic levels (e.g., primary producers, primary consumers, secondary consumers, and decomposers) as interconnected, dynamically evolving networks. Within this framework, nodes represent species or functional groups, while links denote the interactions among them. By analysing network topology (e.g., connectivity, modularity, nestedness, and specialisation) and dynamic properties (e.g., robustness and vulnerability), researchers can gain a more holistic understanding of how invasions perturb systems and how recipient communities respond to such perturbations [49]. Multi-trophic interaction research examines indirect effects and trophic cascades across three or more trophic levels but does not necessarily involve whole ecosystem sampling or quantitative network metrics. By contrast, genuine network-ecology approaches require ecosystem, wide sampling and quantitative evaluation of topological properties such as connectance, modularity, nestedness and specialisation. These metrics can provide deeper mechanistic insights into how biological invasions restructure species interaction webs.

The methodological toolkit for studying multitrophic networks has advanced considerably over the past decade. Traditional approaches, including mesh-based collection, rearing trials, and morphological identification, remain valuable but are labour-intensive and often fail to detect cryptic interactions, such as multiple parasitism or hyperparasitism [50,51]. Molecular techniques, particularly DNA barcoding, DNA metabarcoding, and metagenomics, have revolutionised network construction [52,53,54]. By extracting total DNA from small insect specimens (e.g., parasitoid wasps or lepidopteran larvae) and amplifying marker genes such as mitochondrial COI, high-throughput sequencing platforms enable simultaneous detection of hosts, primary parasitoids, and hyperparasitoids from the same sample [53]. This approach can uncover hidden parasitism, multiparasitism, and superparasitism that are difficult or impossible to detect through conventional rearing and morphological identification, thereby greatly enhancing resolution of food-web structure and biological control potential [52]. For example, Kitson et al. [50] used nested-tagging DNA metabarcoding to characterise the parasitoid community attacking the invasive oak processionary moth *Thaumetopoea processionea* (Linnaeus, 1758) (Lepidoptera: Notodontidae), revealing that one parasitoid species *Carcelia iliaca* (Ratzeburg, 1840) (Diptera: Tachinidae) accounted for 45.7% of parasitism events.

Molecular methods also enable detection of “hidden” mortality, cases where parasitoids kill hosts through feeding or oviposition without completing development (non-reproductive host death). Hepler et al. [55] employed species-specific PCR to detect the presence of the egg parasitoid *Trissolcus japonicus* (Ashmead, 1904) (Hymenoptera: Scelionidae) on egg masses of the brown marmorated stink bug *Halyomorpha halys* (Stål, 1855) (Hemiptera: Pentatomidae). They found that, in addition to causing typical successful parasitism, this introduced parasitoid induced an additional 5.6% egg hatching failure and hidden mortality ranging from 5.4% to 43.2% in eggs of local non-target stink bugs, a finding that challenges conventional pre-release risk assessment frameworks. Furthermore, stable isotope analysis (δ^13^C and δ^15^N) provides complementary information on trophic positioning and resource use within food webs, revealing shifts in dietary strategies and energy flow following invasion [56]. Collectively, these molecular and biogeochemical tools offer powerful means to construct high-resolution food webs and deepen our mechanistic understanding of invasion-driven community reorganisation [53].

Beyond descriptive network characterisation, experimental approaches have been instrumental in linking plant chemical traits to multitrophic interactions. Lin et al. [57] demonstrated that volatiles emitted by native populations of *Cotesia popularis* (Haliday, 1834) (Hymenoptera: Braconidae) induced by herbivory attracted the parasitoid *C. popularis* more strongly than those from invasive populations, indicating that invasive plants can modulate tri-trophic interactions (plant, herbivore, and natural enemy) through volatile signals, thereby promoting invasion success. The release of plant volatiles reflects a balance among multiple selective pressures, including concealment from herbivores, deterrence of omnivorous pests, and recruitment of natural enemies. This study provides key evidence for the evolutionary dynamics of phytochemical defence in the context of biological invasions and underscores the importance of integrating chemical ecology with multitrophic network approaches.

### 3.2. Disruption of Native Plant–Insect Food Web Structure by Biological Invasions

#### 3.2.1. Network Connectivity and Modular Dynamic Reconfiguration

Invasive species profoundly perturb the structure of native plant–insect food webs through the replacement of resident flora and the introduction of novel resources, including invasive plant tissues and exotic insect herbivores, thereby altering food web composition, architecture, and function.

Food web structural metrics provide sensitive indicators of invasion impacts. Connectivity, the proportion of realised to all possible trophic links, can either decrease or increase following invasion, depending on the invader’s functional role and the responsiveness of the recipient community.

Invasion often drives the loss of previously established species or links, thereby eroding network connectivity. Rizali et al. [58] demonstrated that increasing oil palm plantation age (from 3 to 18 years) not only altered plant community composition but also significantly reduced species richness, link density, and interaction diversity in host–parasitoid food webs, driven by long-term resource substitution. Similarly, habitat fragmentation and homogenisation associated with monoculture systems, often dominated by invasive plants or single crops, can simplify parasitoid–aphid food web structure [59,60]. Conversely, landscape complexity can buffer such simplification: Dong et al. [49] found that a higher proportion of woodland in the surrounding landscape increased the relative abundance of primary parasitoids while suppressing hyperparasitoids, thereby enhancing biological control potential against aphids in greenhouse systems.

Beyond compositional changes, invasions can significantly alter interaction frequencies and functional connectivity without modifying the qualitative presence of links (i.e., the binary network skeleton). Barbosa et al. [61] experimentally reduced the density of a common shrub and found that while no trophic links were lost, weighted connectivity, interaction evenness, and network robustness against secondary extinctions all declined substantially. This finding indicates that shifts in resource abundance alone, even without species extirpation, can indirectly destabilise food webs by reconfiguring energy-flow pathways across trophic levels. Xi et al. [62] reported analogous findings: when the abundance of *Saussurea nigrescens* Maxim. (Asteraceae) was experimentally reduced, generalist and specialist phytophagous flies exhibited divergent responses, generalists shifted to untreated host plants, while specialists increased parasitism rates on remaining hosts, with corresponding changes in parasitoid visitation frequencies. Such responses cannot be explained by neutral processes but instead reflect adaptive foraging behaviour.

An even more intricate phenomenon arises when introduced parasitoids facilitate novel host use by resident species, a “rescue” effect. Moon et al. [63] observed that the native parasitoid *Asobara* cf. *rufescens* (Förster, 1862) (Hymenoptera: Braconidae) develops poorly on the invasive *Drosophila suzukii* (Matsumura, 1931) (Diptera: Drosophilidae) under laboratory conditions, yet successfully parasitises this host in the field. This paradox was resolved by the discovery that another introduced parasitoid, *Leptopilina japonica* (Novković & Kimura, 2011) (Hymenoptera: Figitidae), compromises the immune system of *D. suzukii*, thereby “paving the way” for the native parasitoid to exploit an otherwise refractory resource. This case illustrates that invasion-driven introductions of natural enemies can restructure interaction networks and create novel ecological opportunities for native species.

Finally, chemically mediated trophic interactions play a central role in these reconfiguration processes. Leskey et al. [64] reviewed the use of volatile chemical signals in monitoring and managing invasive insect herbivores, noting that while such approaches have proven successful in some systems, their application is complicated when native and invasive species share similar chemical profiles, leading to cross-attraction and non-target effects. Integrating chemical ecology with network analysis thus offers a promising avenue for understanding and predicting the cascading consequences of invasions on food web structure and function.

#### 3.2.2. Loss of Keystone Species and Cascading Disruption of Interaction Chains

Invasive plants frequently establish themselves as novel dominant species, directly impacting insect communities that depend on native flora. A striking example comes from Hawaii, where the invasive gall wasp *Quadrastichus erythrinae* (Kim, 2004) (Hymenoptera: Eulophidae) has inflicted severe damage on trees of the genus *Erythrina* L. (Fabaceae), pushing the endemic *Erythrina sandwicensis* O.Deg. (Fabaceae) to the brink of extinction. This decline extends far beyond a single tree species, triggering the collapse of an entire food web of insect herbivores and their natural enemies, including the specialist parasitoid *Eurytoma erythrinae* (Gates & Delvare, 2008) (Hymenoptera: Eurytomidae), all of which depend on the tree [65].

Concurrently, invasions can also foster the formation of novel trophic links between native species and herbivores or natural enemies, resulting in the assembly of “novel” food webs. These newly established links may exhibit high degrees of specialisation. For instance, the recent invasion of *Aleurocanthus spiniferus* (Quaintance, 1903) (Hemiptera: Aleyrodidae) in Italy was found to be attacked by a suite of native and unintentionally introduced parasitoids, forming a new host–parasitoid complex [66]. Similarly, following its inadvertent introduction from China to Spain, the specialist parasitoid *Trioxys liui* (Chou & Chou, 1993) (Hymenoptera: Braconidae, Aphidiinae) established a novel tri-trophic association (parasitoid–aphid–bamboo) on local bamboo species [67]. The formation of such novel links may contribute to ecosystem stability by providing alternative pathways for energy flow and biological control.

Systematic syntheses indicate that the responses of native insect communities to invasive plants are governed by multiple interacting factors. Invasive plants can alter the performance of native herbivores and their natural enemies (parasitoids and predators), potentially driving host shifts in both herbivores and their antagonists. Moreover, invasive plants modify the chemical complexity of habitats through the release of volatile organic compounds, thereby influencing the behaviour of native insects, including herbivores, parasitoids, and pollinators, across multiple trophic levels [68]. These multifaceted effects underscore the need for integrated, community-level assessments that move beyond pairwise interactions to capture the full spectrum of invasion-driven reorganisation in plant–insect food webs.

### 3.3. From Binary Interactions to Network Interactions: Trophic Cascades, Indirect Effects, and Multitrophic Feedback

Traditional invasion research has largely concentrated on direct pairwise interactions between invasive plants and their natural enemies, such as herbivores or soil pathogens. However, ecosystems are woven from complex networks of indirect effects, and the impacts of invasions are often propagated and amplified through these non-trophic pathways. Recognising this is essential for a comprehensive understanding of invasion consequences.

#### 3.3.1. Coupling Mechanisms of Aboveground–Belowground Ecological Pathways

Plants act as “ecological engineers” bridging aboveground and belowground communities. Changes in belowground biota, including soil microorganisms, herbivorous nematodes, and earthworms, induced by invasive plants can feed back directly to the population dynamics of aboveground insects. For example, Oduor et al. [17] demonstrated that over time, *S. canadensis* accumulates soil microorganisms (e.g., mycorrhizal fungi) that are beneficial to its own performance, establishing a positive feedback loop that enhances its competitive ability against native plants for aboveground resources, including responses to insect herbivory. Similarly, Guerrero et al. [18] showed that the invasive *P. elliottii* not only gained an advantage through the loss of specialist nematodes (enemy release) but also altered soil nematode community composition, reducing the abundance and diversity of phytophagous nematodes, thereby creating novel conditions for the establishment of rhizosphere microbial communities in later invasion stages. These studies underscore that understanding why invasive plants successfully replace native species and support (or suppress) particular insect populations requires explicit consideration of positive feedback loops operating through soil biota and their associated microorganisms.

Aboveground–belowground coupling is not limited to plant–soil interactions; plant pathogens can also mediate such effects. Wyckhuys et al. [69] investigated phytoplasma-infected cassava and found that infection altered plant quality, triggering outbreaks of generalist mealybugs, including the invasive *Phenacoccus manihoti* (Matile-Ferrero, 1977) (Hemiptera: Pseudococcidae), and indirectly affecting parasitoid community structure and richness through cascading changes in plant community composition. This illustrates that microbial infections can reconfigure multitrophic interactions by modifying plant traits that serve as resources or cues for both herbivores and their natural enemies.

Furthermore, the rhizosphere and phyllosphere microbiomes of invasive plants play pivotal roles in mediating interactions with insects. These microbial communities can influence herbivore performance and natural enemy attraction by altering plant nutritional status, inducing systemic resistance, and metabolising plant secondary compounds. Concurrently, secondary metabolites released by invasive plants can modify soil chemical properties, creating favourable conditions for their own spread [3]. Collectively, these findings highlight that aboveground–belowground feedbacks are not merely ancillary but are integral to the invasion process, necessitating a holistic, network-based approach that links soil communities, plant physiology, and multitrophic insect interactions.

#### 3.3.2. Indirect Regulation Mediated by Higher-Order Predators and Parasitoids

Within food webs, higher-order predators, including predatory insects, amphibians, birds, and parasitoid wasps, not only regulate herbivore populations but also, through their introduction or loss, can trigger cascading effects that reverberate throughout entire networks. These effects are particularly intricate in the context of biological invasions.

At the first level, the classical case of parasitoids as biological control agents illustrates the power of top-down regulation. Successful introduction of host-specific parasitoids can exert strong suppressive pressure on invasive herbivores, thereby rescuing native plant communities. Wyckhuys et al. [70,71] documented, at a continental scale, that following the introduction of the specialist parasitoid *Anagyrus lopezi* (De Santis, 1964) (Hymenoptera: Encyrtidae), effective control of the invasive cassava mealybug was achieved across extensive cassava-producing regions in Southeast Asia. Biological control restored crop yields by as much as 10 tonnes per hectare, generating substantial social and ecological benefits. Crucially, by alleviating food security pressures and reducing the need for agricultural expansion, this intervention led to a 31–95% reduction in deforestation rates across four Southeast Asian countries. This exemplifies a profound positive impact on ecosystems mediated through a multitrophic (plant–herbivore–natural enemy) cascade.

At the second level, parasitoids can exert asymmetric suppression of host populations through “non-reproductive host mortality”, host death that does not result in successful parasitoid offspring production. Such mortality can arise from host feeding, oviposition-induced abortion, or physical damage inflicted by the parasitoid. Kaser et al. [72] demonstrated through modelling that non-reproductive host mortality can significantly alter population dynamics, generating indirect interspecific effects, including apparent competition, mutualism, or partial commensalism, depending on host suitability and parasitoid constraints such as egg or time limitation. In the case of the invasive stink bug *H. halys*, less suitable non-target native bugs may suffer greater losses than the target host, as they are parasitised while simultaneously facing feeding attacks from introduced parasitoids; unprotected native populations may decline rapidly due to such hidden mortality. Consequently, biological control risk assessments must extend beyond parasitism success (i.e., offspring production) to quantify the cryptic effects arising from non-reproductive host death [55].

At the third level, complex invasions can directly reshape the niches and population dynamics of apex predators by altering community structure. For example, the invasive European catfish *Silurus glanis* (Linnaeus, 1758) (Actinopterygii: Siluridae) occupies a different trophic niche in lakes compared to native pikeperch, exhibiting a stronger reliance on littoral prey and possessing greater PCB bioaccumulation and biomagnification capacity—with increasing body size doubling contaminant levels. By virtue of its limited metabolic excretion pathways, this invader acts as an “amplifier” of pollutant transfer through the food web [73]. Thus, invasion-driven restructuring of food webs can not only affect biodiversity but also lead to the redistribution and amplification of toxic substances across trophic levels.

At the fourth level, predator diversity can enhance the temporal and spatial stability of ecosystems. Rakowski et al. [74] demonstrated in aquatic mesocosms that when two predator species coexist, they exert complementary size-selective control on zooplankton, reducing temporal variability in individual zooplankton communities and significantly dampening fluctuations in primary producer (phytoplankton) biomass. This “diversity–stability cascade” driven by predator complementarity suggests that in biological control, introducing multiple natural enemies with functionally complementary traits may yield more stable suppression than reliance on a single “super-enemy”. Collectively, these multi-level mechanisms underscore the necessity of accounting for higher-order trophic interactions and their indirect, cascading effects when predicting and managing invasion outcomes.

### 3.4. Case Analysis of Network Structure Reorganisation in a Representative Invasion System

The cassava–mealybug–parasitoid system serves as a paradigmatic example illustrating the power of a multitrophic network perspective in understanding invasion dynamics and guiding biological control. In 2008, the invasive mealybug *P. manihoti* invaded Southeast Asia, causing up to 18% yield losses and driving sharp price increases for cassava products. This economic shock directly stimulated large-scale expansion of cassava cultivation in neighbouring countries and triggered significant deforestation, particularly in Cambodia and Laos [70,71]. This sequence clearly demonstrates how an invasive herbivore can indirectly cause severe ecosystem damage—including biodiversity loss and forest degradation—through socioeconomic feedback loops driven by its feeding impacts [71].

Subsequently, in 2009, the introduction of the host-specific parasitoid *A. lopezi* effectively halted this trajectory. The parasitoid achieved parasitism rates exceeding 50–90% across broad areas of Southeast Asia, rapidly suppressing mealybug populations below economic injury levels [70]. This top-down regulation ultimately alleviated pressure on cassava supply, thereby curbing farmer-led deforestation and generating substantial co-benefits for tropical forest conservation [71]. A more nuanced analysis reveals that the success of this system depends on several key trophic interactions:

1. First trophic level (plant): Cassava growth is influenced by phytoplasma infection, which significantly alters the species composition and population dynamics of associated mealybugs, favouring generalist species, including the target invader *P. manihoti* [69]. This indicated that soil-borne microorganisms can indirectly shape competitive outcomes at the second trophic level by modulating host plant physicochemical properties, with generalist herbivores benefiting disproportionately from pathogen-induced changes.
2. Second trophic level (primary consumers): Invasive mealybugs achieved rapid population outbreaks through efficient utilisation of cassava and release from co-evolved natural enemies, a classic enemy release effect.
3. Third trophic level (natural enemy): The introduced parasitoid *A. lopezi* re-established dynamic equilibrium through sustained top-down suppression. Additionally, this tri-trophic system is modulated by hyperparasitism and incidental host attacks. For instance, Kaspi et al. [75] evaluated the parasitoid *Trichogrammatoidea cryptophlebiae* (Nagaraja, 1979) (Hymenoptera: Trichogrammatidae) for control of another invasive pest *Thaumatotibia leucotreta* (Meyrick, 1907) (Lepidoptera: Tortricidae) and found that it parasitised only host eggs within the family Tortricidae (Lepidoptera) (not the target species), thereby enabling focused risk assessment and improving biological control efficiency. Similarly, Ogburn and Walgenbach [76] demonstrated that storage temperature of host eggs significantly influenced parasitism rates and larval development of the parasitoid *Anastatus reduvii* (Howard, 1880) (Hymenoptera: Eupelmidae), highlighting how abiotic factors (e.g., temperature) can cascade through each key trophic interaction.

Beyond these direct trophic linkages, external environmental factors and predictive modelling have proven invaluable. Climatic niche models, such as CLIMEX, have been used to predict spatial overlap between *A. lopezi* and its mealybug host across different climatic regions of Southeast Asia, thereby optimising release strategies [77,78]. The integration of ecoclimatic niche modelling into classical biological control programmes exemplifies how predictive tools can improve the effectiveness of invasive species management.

The cassava case powerfully demonstrates the necessity of moving beyond binary interaction frameworks to embrace multitrophic network integration. Relying solely on the ERH, identifying the absence of natural enemies, represents only an initial step; the combination of plant disease dynamics, socioeconomic drivers, and landscape-scale changes (e.g., deforestation) is what rendered this biocontrol programme a model of socio-ecological success. This case also re-emphasises the imperative of interdisciplinary integration, spanning molecular ecology, community ecology, and socio-economics, to achieve a true “systems shift” in invasion ecology and biological control.

Finally, complementary studies on stink bug–parasitoid systems further underscore the importance of plant–insect interactions in shaping pest and natural enemy dynamics. Falagiarda et al. [79] demonstrated that host plant preference varies markedly among stink bug species: *H. halys* utilised the broadest range of host plants, while *Palomena prasina* (Linnaeus, 1761) (Hemiptera: Pentatomidae) and *Pentatoma rufipes* (Linnaeus, 1758) (Hemiptera: Pentatomidae) showed stronger affinity for specific families such as Sapindaceae and Rosaceae. Parasitism rates also varied with species and plant family: *T. japonicus* primarily parasitised *H. halys*, whereas *Trissolcus cultratus* (Mayr, 1879) (Hymenoptera: Scelionidae) and two *Telenomus* species targeted *P. rufipes* and *P. prasina*, respectively. Spatio-temporal dynamics and host plant associations significantly influenced species distribution and parasitoid occurrence. These findings highlight that incorporating plant diversity into pest management strategies can enhance parasitoid efficacy, reduce pest populations, and contribute to more sustainable agricultural practices. Collectively, these case studies reinforce the central thesis that multitrophic network perspectives—integrating plants, herbivores, natural enemies, and their abiotic and biotic contexts—are essential for both understanding invasion mechanisms and designing effective, enduring biological control programmes.

## 4. Reflection and Application of Biological Control Strategy Driven by Theoretical Integration

### 4.1. Lessons and Limitations of Traditional Biological Control Strategies

#### 4.1.1. Ecological Limitations of Single-Hypothesis-Based Management Approaches

Classical biological control, particularly the introduction of co-evolved specialist natural enemies, has historically been underpinned by the ERH, which posits that invasive species succeed largely because they have escaped suppression by their native natural enemies; reintroducing such enemies (typically parasitoids or predators) is therefore expected to exert top-down regulation on invader populations [13]. This approach has yielded notable successes, as exemplified by the cassava mealybug system discussed above [70,71]. Nevertheless, its application is constrained by several fundamental ecological limitations.

Firstly, the ERH framework often oversimplifies the complexity of natural enemy, host interactions and their context dependence. While the hypothesis assumes that introduced natural enemies will effectively suppress invaders, Abram et al. [13] critically reviewed classical biological control programmes targeting four invasive stink bug species in North America and concluded that the efficacy of egg parasitoids in reducing pest population growth rates may be frequently overestimated, success is far from guaranteed. Similarly, Walsh-Antzak and Erickson [22] demonstrated that parasitoid impacts on the invasive fig fly *Z. indianus* were density-dependent and competitive-context dependent, with no detectable suppression at low host densities. Moreover, the ERH-derived expectation that invasive species should exhibit reduced defensive investment (as predicted by the EICA hypothesis) is not universally supported; in some cases, introduced populations maintain high levels of immune or chemical defence, potentially attenuating the performance of biological control agents in the introduced range [80].

Secondly, traditional risk assessments based on the ERH tend to underestimate the “hidden” or non-lethal effects of introduced parasitoids on non-target species. Standard screening protocols typically focus on whether an introduced natural enemy can complete development on non-target hosts; if development fails, the agent is often deemed low-risk. However, Kaser et al. [72] and Hepler et al. [55] have shown that parasitoids can impose substantial non-reproductive host mortality, through host feeding, oviposition-induced abortion, or physical injury, even when successful offspring production does not occur. In the case of *T. japonicus* introduced for control of *H. halys*, such hidden mortality on eggs of local non-target species ranged from 5.4% to 43.2% [55]. Thus, risk assessments based solely on “developmental completion” criteria are likely to underestimate the ecological footprint of introduced biological control agents.

Thirdly, a narrow focus on the ERH can lead to misguided resource allocation by overlooking landscape-scale and resource-based drivers of invasion dynamics. The ERH implicitly attributes invasion success primarily to the absence of natural enemies, but empirical evidence indicated that plant resource availability and landscape composition can profoundly modulate trophic interaction frequencies. Xi et al. [62] demonstrated that experimental reduction in host plant abundance altered interaction frequencies between herbivores (generalists vs. specialists) and their parasitoids in a non-neutral manner, driven by adaptive foraging rather than passive processes. Similarly, Syahidah et al. [60] showed that the composition of tropical agricultural landscapes significantly structures host–parasitoid food webs, implying that biological control outcomes are contingent on the broader landscape context. Nevertheless, quantifying the contribution of general predators remains challenging due to their opportunistic feeding habits and complex nutrient interactions. We recommend that future studies combine field trials and long-term monitoring to assess the impact of different enemies more rigorously. Therefore, effective management strategies need to combine the perspectives of resource availability and landscape heterogeneity with the introduction of nature-enemy. This requires moving beyond single hypothesis approaches to accommodate the full complexity of multitrophic, multi-scale ecological interactions.

#### 4.1.2. Reassessment of Control Efficacy and Ecological Risk

Given the limitations outlined above, a more comprehensive and critical reassessment of both the success rates and ecological risks associated with classical biological control programmes is warranted. With respect to control efficacy, while numerous historical introductions have achieved considerable suppression under specific ecological conditions, such as at release sites or during early establishment phases, the overall success rate of natural enemy introductions remains strikingly low. Gutierrez et al. [81] advocated for the use of physiologically based demographic models (PBDMs), driven by weather and host–plant dynamics, to conduct ex-ante analyses that improve the predictability of natural enemy performance prior to release. These models integrate physiological and ecological parameters of plants, pests, and natural enemies with climatic data, enabling more accurate forecasts of establishment and impact, and thereby offering robust support for natural enemy selection.

Concerning ecological risk, traditional host-specificity testing conducted under confined cage conditions often provides an overly narrow assessment and must be complemented by broader, landscape-scale evaluations. Melone et al. [66] investigated the parasitoid community associated with the invasive *A. spiniferus* in Italy and discovered that, in addition to the primary introduced species, the community also included unintentionally introduced species, notably *Encarsia smithi* (Silvestri, 1926) (Hymenoptera: Aphelinidae), detected for the first time in Italy. These unintentional introductions may interact synergistically with intentionally released agents, creating complex “multi-species assemblages” whose combined effects on native parasitoids and food-web structure require systematic risk assessment. Similarly, Caron et al. [82] compared two egg parasitoids, *T. japonicus* and *T. mitsukurii*, for pre-emptive biological control of *H. halys* in Australia, emphasising that evaluations must account for competitive interactions with local parasitoids and potential non-target host feeding.

Furthermore, the integration of biological control with other pest management tactics presents additional challenges. Singh et al. [83] assessed the compatibility of insecticides with the larval endoparasitoid *Tetrastichus planipennisi* (Yang, 2006) (Hymenoptera: Eulophidae), a key agent against emerald ash borer, and found that insecticide exposure significantly reduced parasitism rates, oviposition, and adult longevity. This highlights the need for careful integration of chemical and biological control strategies to avoid undermining natural enemy efficacy. Collectively, these studies underscore that a robust reassessment of biological control must move beyond single-species, single-mechanism frameworks to embrace predictive modelling, landscape-scale risk evaluation, and multi-tactic compatibility analysis, thereby enhancing both the safety and effectiveness of future biocontrol programmes.

### 4.2. A Novel Regulatory Framework Based on Multitrophic Network Theory

To overcome the limitations of traditional biological control, we propose an integrative framework grounded in multitrophic network theory that explicitly incorporates trophic cascades, indirect effects, and landscape context.

#### 4.2.1. Conservation and Resilience Enhancement of Local Food Web Integrity

Biological control should not rely solely on the introduction of novel natural enemies; rather, priority should be given to conserving and enhancing the resistance and resilience of local food webs, primarily through the protection or restoration of habitat diversity. Accumulating evidence indicates that complex landscapes, characterised by a higher proportion of semi-natural habitats and woodlands, can stabilise ecosystem functioning. Dong et al. [49] demonstrated that increasing woodland cover in the landscape surrounding greenhouses enhanced the abundance of primary parasitoids while suppressing hyperparasitoids, thereby improving biological control potential against aphids. Similarly, Yang et al. [84] found that landscape heterogeneity influences the diversity of both parasitoids and hyperparasitoids, which in turn modulates overall biocontrol efficacy. The level of landscape management thus directly affects the functional realisation of food web services [85]. While crop-dominated landscapes may exhibit increased network connectivity, such connectivity is often accompanied by a predominance of generalist or opportunistic interactions that do not necessarily translate into effective pest suppression [60]. In contrast, moderately connected and patch-diverse landscapes support more complex food web structures and higher levels of ecological service provision [59]. Landscape heterogeneity and multi-trophic biodiversity boost ecosystem resistance and resilience against biological invasions. Collectively, these findings underscore that conserving landscape heterogeneity and local food-web integrity offer a foundational strategy for sustainable biological control: this approach reduces reliance on risky exotic introductions while strengthening the natural regulatory capacity of agroecosystems.

#### 4.2.2. Precision Utilisation Strategies of Higher Trophic Levels

Beyond conservation, the proactive introduction or augmentation of functionally complementary higher trophic levels, comprising distinct parasitoid guilds, predatory insects, and even amphibians, represents a promising avenue for enhancing biological control. Rakowski et al. [74] demonstrated in aquatic mesocosms that two predatory hemipterans achieved complementary feeding through differential size-selective predation on zooplankton, jointly stabilising phytoplankton biomass, a clear illustration of the functional benefits conferred by multi-predator combinations. Drawing a parallel to this paradigm, within terrestrial ecological systems, the inoculative release of functionally complementary natural enemies, exemplified by the paired application of egg parasitoids and larval parasitoids, is predicted to generate broader-spectrum and more temporally stable regulation of target pest densities relative to single-agent-dependent control tactics.

In addition to direct introductions, an indirect but potentially powerful strategy involves facilitating the recruitment or “rescue” of native natural enemies, enabling them to exploit previously inaccessible invasive resources. Moon et al. [63] documented a remarkable case in which the introduced parasitoid *L. japonica* weakened the immune system of the invasive host *D. suzukii*, thereby creating a window of opportunity for native parasitoids to successfully attack this otherwise refractory host. This finding suggests that introduced natural enemies can serve not only as direct control agents but also as “enablers” that liberate native parasitoids constrained by host immune defences, resulting in a more potent and synergistic joint suppression effect. Such indirect facilitation underscores the importance of considering community-level interactions and cross-species compatibility when designing introduction programmes. A precision-based strategy, one that explicitly accounts for functional trait complementarity, immune-mediated interactions, and ecological fitting, could thus unlock novel pathways for enhancing biological control outcomes while minimising unintended ecological disruptions.

#### 4.2.3. A Targeted Priority Assessment Framework Integrating Multi-Hypothesis Collaboration

A more comprehensive risk assessment must extend beyond conventional evaluations of parasitism success to incorporate the following dimensions:

1. Non-reproductive host mortality (hidden effects): Molecular detection tools, such as the species-specific PCR approach employed by Hepler et al. [55], should be routinely applied to quantify hidden mortality inflicted on non-target organisms by both introduced and native parasitoids and predators. Furthermore, the modelling framework developed by Kaser et al. [72] can be used to predict whether such non-reproductive mortality may trigger indirect competition or cascade effects at the community level.
2. Multi-trophic cascade effects: Introduced parasitoids not only affect their direct hosts but can also generate ripple effects through hyperparasitoids at higher trophic levels or via host plants at lower levels [49,84]. Effective biological control planning must anticipate both positive and negative outcomes of such cascades. For example, while biological control of ragweed (*A. artemisiifolia*) through introduced natural enemies successfully suppressed the target plant, it also triggered “secondary invasion” by other invasive species in many plots [48]. Future risk assessments and management programmes must explicitly account for these multi-trophic repercussions.
3. Integrative evolutionary and ecological dynamics: Screening potential natural enemies should incorporate predictions from the EICA and NWH frameworks, evaluating the defensive traits and adaptive evolutionary potential of target plants. If an invasive plant exhibits strong EICA-type responses, rapidly reallocating resources from defence to growth and reproduction, reliance on one or two natural enemies may prove insufficient. In such cases, the introduction of multiple functionally complementary natural enemies, combined with additional management measures such as habitat interventions, may be necessary. Ecological niche models (e.g., CLIMEX) provide a valuable tool for identifying optimal release sites for natural enemies, thereby enhancing establishment success and suppression efficacy [77]. Collectively, this multi-faceted, hypothesis-informed priority assessment framework offers a more robust foundation for evidence-based biological control decisions.

Synthesising the three foregoing modules: conservation of local food-web resilience, precision deployment of higher trophic level components, and multi-hypothesis informed priority assessment, we propose an operational template oriented towards invasive plant–insect interaction research and associated biological control practice (Table 2). This workflow can be simplified or adjusted according to practical constraints, invasion stages, spatial scales and management objectives.

### 4.3. Technological Innovations Enabling Precision Monitoring and Predictive Intelligence

#### 4.3.1. Application of DNA Metabarcoding in High-Throughput Interaction Network Analysis

Theoretical advances in invasion ecology and biological control require commensurate methodological innovation. Recent developments in molecular ecology and computational modelling have provided unprecedented technological empowerment for implementing multitrophic network perspectives in biocontrol practice. Among these, DNA metabarcoding and other high-throughput sequencing approaches have become indispensable tools for disentangling complex trophic interactions and are increasingly essential for evidence-based biological control.

Construction of cryptic food webs: Traditional rearing-based approaches can only capture a limited subset of trophic interactions, often missing cryptic parasitism, multiparasitism, or hyperparasitism. DNA metabarcoding enables the simultaneous extraction, amplification, and sequencing of total DNA from mixed samples (such as host insects, soil arthropods, or aquatic organisms), thereby revealing a vast array of interactions in a single experiment [53]. Slusher et al. [52] employed DNA metabarcoding to successfully unravel the aphid–parasitoid–hyperparasitoid network on pecan, discovering that up to eight hyperparasitoid species attack a single parasitoid species simultaneously, providing molecular evidence for why single-agent biological control often fails.

Non-target risk assessment: As discussed previously, Hepler et al. [55] used species-specific PCR to quantify hidden mortality inflicted by introduced parasitoids on non-target host eggs. Molecular methods offer high sensitivity and efficiency in detecting such “traceless” mortality, including host feeding and non-reproductive death, substantially improving the accuracy and comprehensiveness of risk assessments compared to conventional rearing trials [54].

Large-scale food web dynamics tracking: By combining DNA barcoding, metabarcoding, and molecular tagging techniques (e.g., nested tagging), researchers can perform large-scale, high-resolution food web analyses across broad spatial and temporal scales. This enables tracking of how introduced natural enemies disperse across landscapes, form colonisation networks, and establish trophic linkages with resident species [50]. These approaches offer an unprecedented capacity to evaluate the success rates and ecological impacts of biological control at regional scales, thereby facilitating better-informed decision-making in invasive species management.

Collectively, these molecular tools not only enhance the resolution of interaction networks but also enable proactive, predictive risk assessment, transforming biological control from a largely reactive discipline into a data-driven, precision-oriented enterprise.

#### 4.3.2. Scenario Prediction Through Dynamic Network Models and Invasion Forecasting

Modeling techniques, especially the combination of network analysis models and population dynamics models, provide a powerful tool for us to predict the long-term effects of biological control:

Niche modelling: Nidia et al. [77] employed the MaxEnt algorithm to predict the potential overlapping distribution of the invasive cactus moth *Cactoblastis cactorum* (Berg, 1885) (Lepidoptera: Pyralidae) and its parasitoid *Apanteles opuntiarum* (Martínez & Berta, 2012) (Hymenoptera: Braconidae) across North America, thereby guiding optimal parasitoid release sites. Other ecoclimatic niche models, such as CLIMEX, have been widely used to identify climatically suitable provenances for natural enemy exploration and to inform release strategies [78]. Extending this approach to future climate scenarios, Cheng et al. [86] combined MaxEnt and CLIMEX to project the potential distributions of the invasive eulophid *Ophelimus maskelli* (Ashmead, 1900) (Hymenoptera: Eulophidae) and its natural enemy *Closterocerus chamaeleon* (Girault, 1922) (Hymenoptera: Eulophidae), providing a critical basis for long-term monitoring and adaptive management under climate change. However, CLIMEX and similar tools cannot account for all local-scale conditions, such as microclimate, soil heterogeneity, or management history, and should therefore be complemented by field validation and adaptive management approaches. Looking ahead, machine-learning-assisted forecasting that integrates multi-omics data is expected to become increasingly prevalent, enabling predictions of evolutionary trajectories and interaction intensities within invasive plant–insect systems.

Population dynamics and food-web models: Gutierrez et al. [81] applied a physiologically based demographic model to retrospectively analyse the successful biological control of cassava mealybug, demonstrating that such models can predict natural enemy efficacy ex ante, thereby offering a pathway to improve the historically low success rates of classical biological control programmes. Complementing this, Gao et al. [87] used a spatially explicit pair-approximation model to quantitatively examine how release timing, initial predator density, and spatial aggregation influence predation-driven invasion suppression. Their results indicated that earlier releases and greater predator preference for the invasive target yielded superior control outcomes, providing mathematical guidance for precision deployment of natural enemies. By integrating empirical data on multitrophic network structure (e.g., connectivity, specialisation) and function (e.g., biocontrol efficiency), these modelling frameworks collectively enable the development of more predictive and adaptable management strategies capable of coping with complex and dynamically changing environments.

Integrating the theoretical syntheses and technological innovations discussed above, we propose a novel biological control framework grounded in multitrophic network theory (Figure 2). This framework hierarchically integrates climate/global change drivers, competitive interactions between invasive and native plants, herbivore–natural enemy networks, and feedback regulation by soil microbial communities, while contrasting the essential differences between traditional single-hypothesis strategies and the novel integrated approach. As illustrated in Figure 2, molecular tools (DNA metabarcoding, metagenomics) and multi-scale models (niche models, food-web models, physiologically based demographic models) permeate the entire chain of network construction, risk prediction, and efficacy evaluation.

## 5. Current Knowledge Boundaries and Future Perspectives

### 5.1. Systematic Deep Integration of Multidisciplinary Approaches

Future research in invasion biology and biological control must move decisively towards interdisciplinary integration, encompassing at least the following dimensions.

Firstly, the synergy between evolutionary biology and ecology is paramount. Population genomics and evolutionary ecological approaches can elucidate rapid adaptive responses of invasive plants or pests to introduced natural enemies, including defence reallocation and immune system adjustments, over ecologically relevant timescales. For instance, Selechnik et al. [80] demonstrated that expression patterns of immune-related genes (notably those involved in inflammation and cytotoxicity) exhibited complex, nonlinear relationships with natural enemy density in invasive cane toads, revealing rapid population-level genetic dynamics driven by enemy pressure. This finding cautions that even following successful natural enemy introduction, invasive species may evolve novel defence strategies that attenuate long-term control efficacy. Thus, evolutionary dynamics must be explicitly incorporated into adaptive management frameworks. While this review focuses primarily on plant–insect interactions, similar ecological and evolutionary mechanisms may operate in plant–mite systems. Mites (Acari) are among the most diverse and abundant arthropod herbivores, and many species form intimate associations with invasive plants. For example, De Giosa et al. [88] investigated the eriophyoid mite *Acalitus simplex* (Flechtmann & Etienne, 2002) (Acari: Eriophyidae) associated with invasive *Ruellia simplex* C. Wright (Lamiales: Acanthaceae) in Florida. This oligophagous mite induces erineum galls on host tissues and has considered a prospective biological-control agent for managing invasive *R. simplex* in its introduced range. Such plant–mite systems provide valuable opportunities to test whether core invasion hypotheses, including the ERH, the NWH, and the EICA, also hold for non-insect herbivores. Predatory mites (Acari: Phytoseiidae) represent another critical component of invaded multitrophic food webs, whose fitness and population performance are strongly modulated by host-plant characteristics as well as herbivorous-prey availability. McMurtry et al. [89] established a framework that offers a valuable analytical tool for disentangling predatory, mite functional roles within invaded plant–mite–natural-enemy interaction networks, as well as for evaluating shifts in prey resources and microhabitat conditions driven by invasive plants. Future studies should expand multitrophic-network investigations to encompass both herbivorous mites and their acarine natural enemies. Such efforts will improve the generalisability of invasion-ecological theory and broaden the practical application scope of biological-control strategies.

Secondly, the integration of molecular, ecological, and environmental sciences is essential for capturing the full complexity of invasion processes. Gut microbiome profiling, elucidating how commensal bacterial communities affect host fitness and behaviour [28], alongside plant endophyte and phyllosphere microbiome analyses, should be embedded within food-web research. Nakamura et al. [37] found that invasive populations of *A. crenata* harboured higher abundances of foliar pathogenic fungi in their introduced range compared to the native range, suggesting that enemy release or chemical intervention may reshape the phyllosphere microbiome, thereby modulating subsequent interaction strengths. Furthermore, molecular tools can trace the transmission pathways of parasites between invaders and native hosts. Bartolomé et al. [21] revealed that the invasive hornet *V. velutina* shares parasitic pathogens with native bees, implying horizontal transmission via shared food-web pathways (e.g., predation or feeding) that may break down parasite, host barriers between native and invasive species. Collectively, these multidisciplinary approaches, spanning genomics, microbial ecology, and environmental chemistry, offer unprecedented opportunities to unravel the mechanistic underpinnings of invasion success and biological control outcomes, and should be prioritised in future research agendas.

### 5.2. Long-Term Dynamic Monitoring and Global Multi-Scale Comparative Studies

Most current research remains confined to greenhouse experiments or short-term field trials, with a notable scarcity of multi-year observational data under natural conditions. Studies examining multi-trophic interactions in field settings are particularly limited. This temporal constraint severely hampers our understanding of invasion trajectories and the efficacy of biological control interventions, which are often evaluated within project-based timeframes of only a few years. Korell et al. [4] demonstrated that positive feedback effects of the invasive plant *S. canadensis* on its associated natural enemies did not manifest until six years after establishment, underscoring the delayed nature of such interactions. Similarly, Rizali et al. [58] observed that the host–parasitoid network in oil palm plantations became markedly simplified only after 18 years of plantation age, a pattern detectable exclusively through long-term monitoring. These findings highlight the urgent need to establish sustained ecological observation networks (spanning at least 5–10 years) capable of tracking: (1) the survival, dispersal, and establishment of introduced natural enemies; (2) potential rebounds of invasive plant populations or the occurrence of secondary invasions following successful control [48]; and (3) the reconfiguration of local to regional food webs, including the emergence of novel linkages or the loss of existing interactions.

Equally critical are global-scale comparisons across continents and latitudes. The invasion history, available natural enemy pools, and environmental contexts vary substantially among regions, leading to markedly different outcomes for a given invasive species [30,90]. For instance, Narimanov et al. [90] found that the invasive spider *Mermessus trilobatus* (Emerton, 1882) (Araneae: Linyphiidae) was preyed upon at twice the rate of a sympatric native spider by a generalist predator in Europe, indicating that the ERH does not hold in this system. Similarly, Mabey et al. [91] found no support for ERH in their study of invasive brown seaweeds. These cases suggest that generalised, globally applicable explanations for invasion success are unlikely; instead, cross-regional comparative studies are essential to disentangle primary and secondary invasion mechanisms across different ecosystems. Furthermore, public perceptions and social acceptance of biological control, particularly regarding animal welfare concerns associated with certain agents (e.g., parasitoids used against vertebrates), represent additional challenges for global programmes. Effectively communicating scientific expectations and aligning management strategies with societal values will be crucial for the long-term success of biocontrol initiatives [92].

Comparative studies between paired populations from native and introduced ranges remain insufficient, particularly in their parallel application across multiple invasive populations. Future research should prioritise long-term field monitoring and manipulative experiments within standardised observation networks that encompass both native and multiple invaded sites, thereby generating longitudinal interaction data across broad spatial and temporal scales. Notably, formal quantitative network-ecology studies focusing on invasive-plant associated systems are still relatively scarce. Most existing published work remains limited to descriptive assessments of multi-trophic interactions. Therefore, future invasion-biology research should strive to move beyond documenting pairwise or qualitative multi-trophic effects, and adopt quantitative network tools to characterise how invasion reshapes whole interaction webs.

### 5.3. Evolutionary Trajectories of Interaction Networks Under Global Environmental Change

Climate change presents an inescapable challenge for invasion research and biological control. Rising temperatures, altered precipitation regimes, and increased frequency of extreme weather events will profoundly perturb plant–insect–natural enemy interactions across multiple trophic levels. However, studies examining how global change drivers, such as elevated CO_2_, warming, and nitrogen deposition, synergistically modulate invasive plant–insect dynamics remain extremely scarce. Future research must prioritise interaction predictions under multi-factor global change scenarios.

1. Shifts in natural enemy efficacy: Lann et al. [47] systematically reviewed the effects of temperature variation on phenotypic plasticity and adaptive strategies of parasitoids and their hosts, concluding that under projected climate warming, phenological mismatches and spatial niche separation between parasitoids and their target pests may render currently effective natural enemies unable to suppress pest populations in the future. For instance, parasitoids with limited heat tolerance, particularly those active beneath tropical canopies, may be among the first to lose functionality under heat stress [93].
2. Range expansion and invasion facilitation: Climate change will amplify the frequency and intensity of plant invasions, imposing new demands on biological control programmes. The combined effects of climate change and plant invasions have already destabilised ecosystem networks, reduced biodiversity, and triggered cascading socio-ecological consequences [94]. Ecoclimatic niche models such as CLIMEX project that many invasive species will expand their distributions towards mid- and high-latitude regions [78]. To mitigate these risks, predictive distribution models should be constructed proactively, and potential natural enemy populations from warmer source regions should be identified and conserved in advance.
3. Disruption of chemical communication: Beyond direct thermal effects, climate change can subtly rewire trophic interactions by altering plant chemical phenotypes. Munawar et al. [45] demonstrated that heat stress significantly modified the volatile profile of potato plants, enhancing repellency to the pest *Phthorimaea operculella* (Zeller, 1873) (Lepidoptera: Gelechiidae) while simultaneously increasing attractiveness to its parasitoids. This finding illustrates that climate change operates not merely through temperature elevation but also through subliminal perturbations of the chemical information channels that underpin plant–herbivore–parasitoid interactions, thereby influencing the ultimate efficacy of biological control.

Collectively, these multifaceted impacts underscore the urgent need for integrative, cross-disciplinary research that couples climate projections with multitrophic network models to anticipate and manage the evolving dynamics of invasions in a warming world.

## 6. Conclusions

This review has critically re-examined three classical hypotheses, the Enemy Release Hypothesis (ERH), the Novel Weapons Hypothesis (NWH), and the Evolution of Increased Competitive Ability (EICA) hypothesis, within the framework of plant–insect interactions in biological invasions. We conclude that these hypotheses are not mutually exclusive or universally applicable; rather, they represent interconnected, context-dependent ecological processes whose relative importance varies with invader identity, invasion stage, community composition (e.g., natural enemy assemblages), and environmental conditions (e.g., climate and nutrient availability). No single hypothesis adequately captures the complexity of real-world invasions; their integration is essential for a more nuanced mechanistic understanding.

Crucially, we advocate for embedding plant–insect interactions within a broader multitrophic network perspective. Invasions do not simply alter pairwise plant–herbivore relationships; they profoundly reorganise complex above- and belowground food webs via cascading indirect effects spanning plants, soil biota, herbivores, natural enemies, hyperparasitoids, and microorganisms. This network lens reconciles apparent contradictions between classical predictions and empirical observations by accounting for previously neglected indirect pathways, while revealing that changes in the abundance of even a single species can trigger far-reaching and often unexpected consequences across the ecosystem.

Building on this theoretical integration, we propose a novel biological control framework that moves beyond the traditional “introduction-release-effect” model. This framework prioritises: (1) conserving and restoring local food web complexity and stability as a primary defence; (2) deploying functionally complementary natural enemies or facilitating the recruitment of resident antagonists through higher-trophic-level regulation; and (3) incorporating hidden host mortality and non-target indirect effects into comprehensive risk assessments. The application of molecular tools, including DNA metabarcoding and metagenomics, alongside multi-scale modelling approaches (e.g., niche models, food-web models, and physiologically based demographic models), provides robust technical support for operationalising this integrated framework.

Looking forward, significant opportunities and challenges lie ahead. We call for interdisciplinary, long-term, and large-scale observational and experimental studies that capture the dynamic responses of multitrophic interactions and management interventions in the context of global climate change. Only through such systematic and integrative approaches can invasion ecology translate its theoretical advances into precise, effective, and sustainable strategies for preventing and managing biological invasions, thereby making tangible progress in safeguarding biodiversity and sustaining human well-being.

## Figures and Tables

**Figure 1:**
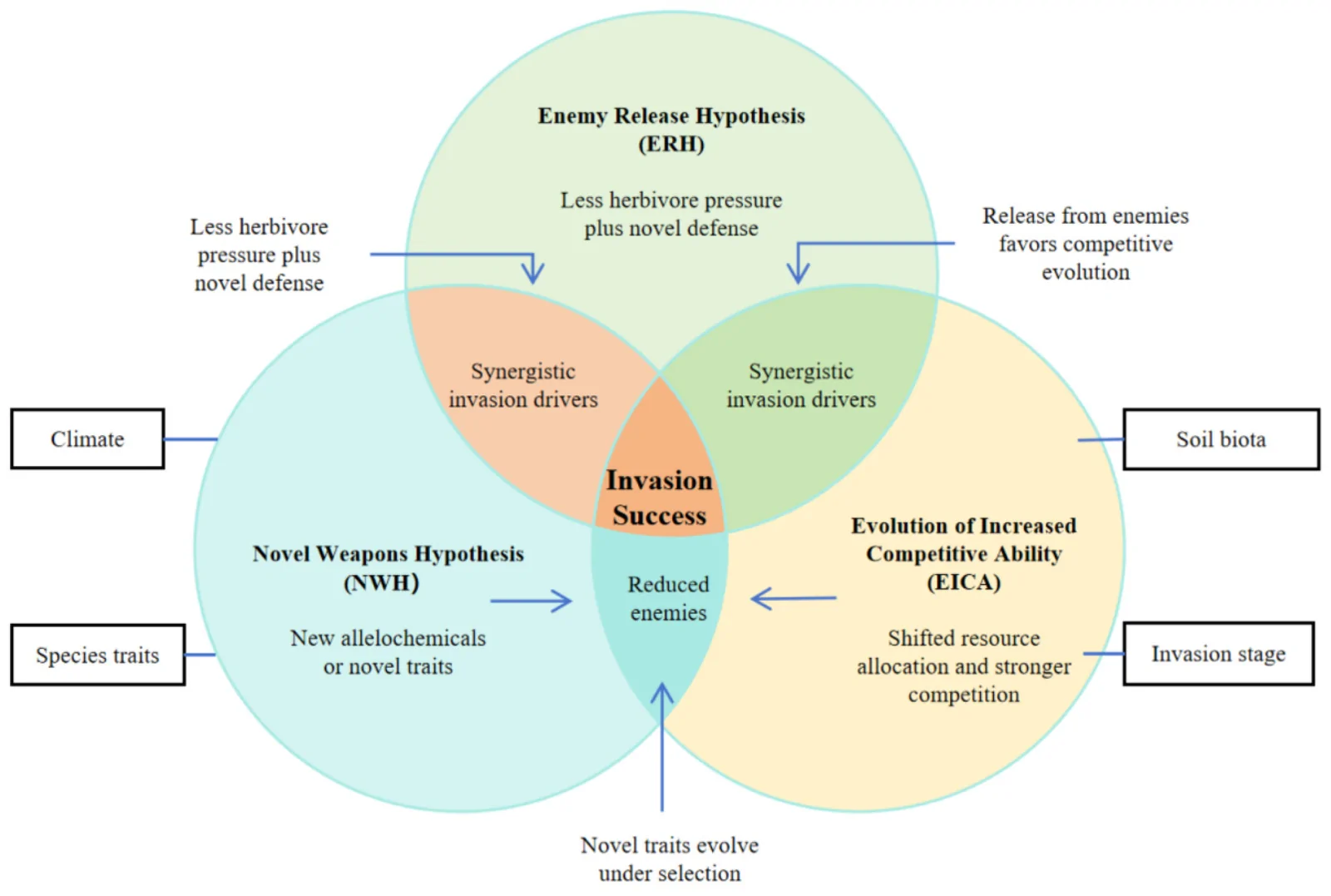
Synergistic relationships and integrative framework of three classical hypotheses in invasion ecology.

**Figure 2:**
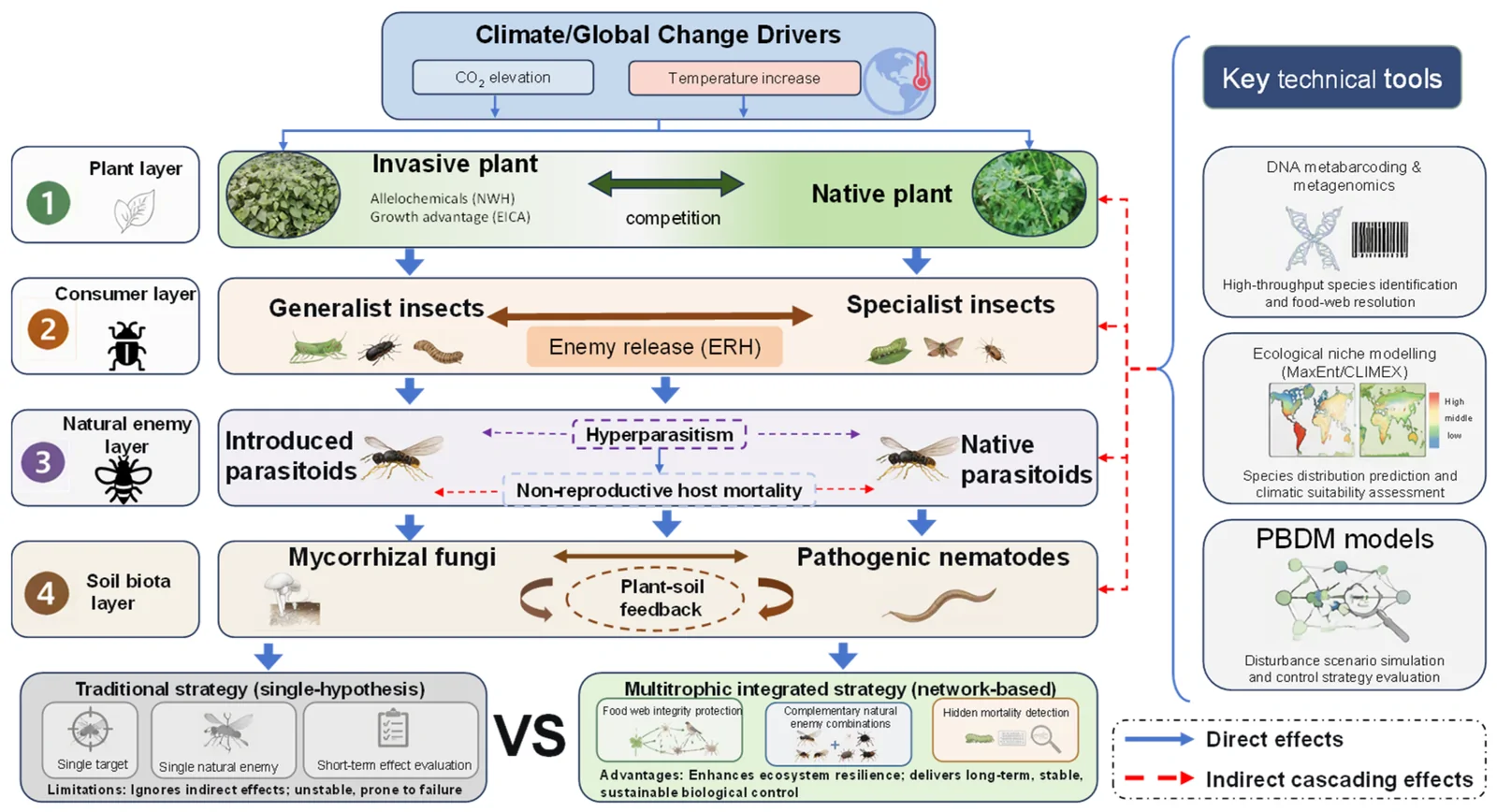
Multitrophic network integration framework and novel biological control strategies. The diagram integrates the Enemy Release Hypothesis (ERH), Novel Weapons Hypothesis (NWH), and Evolution of Increased Competitive Ability (EICA) with multitrophic network theory across four trophic tiers: (1 (green) Plant layer, 2 (orange) Consumer layer, 3 (purple) Natural enemy layer, and 4 (brown) Soil biota layer), regulated by climate drivers (CO_2_ and temperature). Solid blue arrows indicate direct effects; dashed red arrows indicate indirect cascading effects. Green double-headed arrows indicate competition between invasive and native plants. Orange double-headed arrows indicate ERH between generalist and specialist insects. Purple dashed arrows indicate hyperparasitism, and red dashed arrows (middle) indicate non-reproductive host mortality between introduced and native parasitoids. Brown circular arrows indicate plant-soil feedback between mycorrhizal fungi and pathogenic nematodes. Side panels list key methodological tools (DNA metabarcoding, niche modelling, PBDM). The bottom panel contrasts traditional single-hypothesis strategies (grey) with the proposed multitrophic integrated strategy (colour), which prioritises food-web integrity, complementary natural enemies, and hidden mortality detection for sustainable biological control. PBDM, physiologically based demographic models.

**Table 1:** Core predictions, empirical support, and key limitations of three classical invasion hypotheses.

Hypothesis	Core Prediction	Key Limitations/ Controversies	Association with Plant–Insect Interactions	References
Enemy Release Hypothesis (ERH)	Escape from co-evolved specialist natural enemies (insect herbivores, pathogens) in the native range, allowing reallocation of defence resources to growth and reproduction, thereby enhancing competitive advantage	Approximately half of introduced species do not show detectable enemy release Release strength shows no significant correlation with time, temperature, precipitation, or altitude Invasive plants may accumulate generalist enemies, causing “enemy spillover” that disproportionately harms native species Release effects are highly density-dependent and context-dependent ERH focus on specialist enemies may overlook the role of generalist granivores and omnivores	Direct reduction in herbivore pressure alters competitive hierarchies between invasive and native plants; reduced structural defences (leaf density, cellulose, lignin) may indirectly affect herbivore feeding preferences and performance	[7,20,23,24]
Novel Weapons Hypothesis (NWH)	Production of allelochemicals (“novel weapons”) to which native species lack evolutionary exposure, suppressing native plants, insects, and soil biota	Allelopathic effects are strongly concentration-dependent; high-concentration laboratory extracts may not reflect field soil conditions Soil microbes can gradually adapt to and degrade allelochemicals, weakening effects over time Confusion over definitions and insufficient empirical evidence; rigorous field experiments are needed Glucosinolates of *Alliaria petiolata* (M.Bieb.) Cavara & Grande (Brassicaceae) may not be the key novel weapon driving its invasion Specialist beetle larvae *Lema daturaphila* (Kogan & Goeden, 1970) (Coleoptera: Chrysomelidae) co-opt host-plant alkaloids for defence against parasitoids, revealing that allelochemicals can be exploited by herbivores	Allelochemicals directly influence insect feeding preferences, development, and survival through toxicity or antifeedant activity; volatile compounds may affect host location and oviposition behaviour; soil-mediated effects on decomposers indirectly affect nutrient availability for herbivores; in some cases, insects may co-opt plant toxins for their own defence	[15,31,34,35,36]
Evolution of Increased Competitive Ability (EICA) Hypothesis	Escape from specialist natural enemies reduces selection for costly defences, allowing reallocation of resources to growth and reproduction, thereby enhancing competitive ability	• Meta-analysis found that EICA predictions on defence and competitiveness are not widely supported across species • Invasive plants may shift to “qualitative defence” (changing toxin composition) rather than simply reducing total investment • Defence–growth trade-off may be highly context-dependent • *Solidago altissima* L. (Asteraceae) in invaded Japan experienced higher herbivore densities (*Corythucha marmorata* (Uhler, 1878) (Hemiptera: Tingidae)) than in native USA, with induced resistance rather than reduced defence	Altered defence–growth resource allocation indirectly affects herbivores and their natural enemies through changes in plant nutritional quality and chemical profiles; reduced structural defences may increase palatability to generalist herbivores; qualitative shifts in chemical defence may differentially affect specialist vs. generalist herbivores, reshaping herbivore community structure	[20,39,40,41,43]

**Table 2:** Operational template for multitrophic-network-based biological control research on invasive plant–insect systems.

Stage	Objective	Key Methods	Expected Output
1. Baseline network construction	Reconstruct interaction networks in invaded and native ranges	Field sampling; DNA metabarcoding; gut-content analysis; stable isotopes	High-resolution food-web topology
2. Identification of key nodes and interaction strengths	Determine which plant, herbivore, and parasitoid species are central to network stability and invasion dynamics	Network metrics (centrality, specialisation, robustness); interaction-strength quantification	Ranking of keystone species and critical trophic links
3. Selection of complementary natural enemies	Identify functionally complementary predator combinations for suppression	Trait-based complementarity analysis; niche overlap assessment	Candidate agent combinations with minimal niche overlap and maximal suppression potential
4. Assessment of hidden mortality and non-target effects	Quantify non-reproductive host mortality and indirect effects on non-target species	Species-specific PCR; molecular gut-content analysis; field exclusion experiments	Risk profiles for each candidate natural enemy; quantification of hidden ecological costs
5. Predictive modelling and adaptive management	Forecast control efficacy under current and future climate scenarios	Ecological niche models (MaxEnt/CLIMEX); PBDM; food-web dynamic models	Spatially explicit release plans; climate-adjusted management recommendations; monitoring protocols

Note: This process combines food web protection, natural enemy functional complementarity, multi-hypothesis-driven risk assessment, and predictive adaptive management considerations. Individual stages can be simplified or adjusted according to actual restrictions, invasion stages, spatial scales, and on-site management.

## Data Availability

No new data were created or analyzed in this study. Data sharing is not applicable to this article.
